# Endodontic Rotary Systems: Comparison between Gentlefile and Pro Taper Universal for removal of *Enterococcus faecalis*

**DOI:** 10.4317/jced.60147

**Published:** 2023-04-01

**Authors:** Mostafa Godiny, Bahare Mohammadi, Maryam Norooznezhad, Maryam Chalabi

**Affiliations:** 1 DDS, Endodontics Department, School of Dentistry, Kermanshah University of Medical Sciences, Kermanshah, Iran; 2 DMD, Medical Biology Research Center, Health Technology Institute, Kermanshah University of Medical Sciences, Iran; 3 DMD, Oral and Maxillofacial Radiology, Dental Branch, Islamic Azad University, Tehran, Iran; 4 Ph.D., School of Dentistry, Kermanshah University of Medical Sciences, Kermanshah, Iran

## Abstract

**Background:**

As a resistant bacterium species in infected root canals, *Enterococcus faecalis* needs to be removed in any endodontic treatment. So, we aimed to compare the effectiveness of two rotary systems, Gentlefile and Pro Taper Universal, in removing *Enterococcus faecalis* (*E. faecalis*) from the infected root canal system.

**Material and Methods:**

Forty single-root premolar teeth were collected and randomly divided into two groups: Gentlefile (n=18) and Pro Taper Universal (n=18). In addition, four teeth were used as a negative control. The root canals were prepared and infected with E. faecalis and incubated for 4 weeks. Samples were obtained from the root canal immediately before and after instrumentation. A reduction in bacteria was determined by the colony count method.

**Results:**

Colony numbers of E. faecalis were significantly different before and after instrumentation in all groups (*p*<0.001). Furthermore, Gentlefile group illustrated a higher percentage of bacterial reduction (96.1%) compared to Pro Taper Universal group (90%). Accordingly, Gentlefile group was found to be significantly (*p*<0.001) more effective in decreasing bacterial populations than Pro Taper Universal group.

**Conclusions:**

Although both rotary systems were highly effective in bacterial reduction from root canals, Gentlefile demonstrated better bacterial reduction percentage from root canals than Pro Taper Universal.

** Key words:**Enterococcus faecalis, Gentlefile, Pro Taper Universal, Rotary systems.

## Introduction

An issue faced by endodontists is to eliminate the bacteria and their products during root canal instrumentation (1). On the other hand, there are factors that could lead to persistent root canal infections such as all instruments and preparation techniques used for root canal treatment, debris, and bacteria extrusion. *Enterococcus faecalis* (*E. faecalis*), a gram-positive facultative anaerobic coccus, is the most commonly isolated bacteria from teeth received endodontic post-treatments (2). According to certain studies, entombed *E. faecalis* can survive in poor-nutrient of treated root canals for a long time (3,4). On the other hand, these microorganisms are resistant to conventional treatments and therefore, contribute to the development of apical periodontitis (5).

Recently, diverse rotary systems such as Pro Taper and Gentlefile have been introduced for apical extrusion of debris. By having good flexibility, super-elasticity, and shape-memory, Nickel-Titanium (NiTi)-based instruments, such as Pro Taper, are commonly used in endodontic treatment (6). NiTi rotary files, as a standard technique, are rapid and fracture resistance with centered preparation results (1). Therefore, these instruments have become quite popular in practice. Compared with the conventional ProTaper System, NiTi-based ProTaper Universal files have a progressive taper design which improves both flexibility and cutting efficiency. In addition, this system can reduce torsional loading, canal transportation, and cyclic fatigue of the file (7). Gentlefile is a rotary system made consisted of ultra-flexible stainless steel braided wires with an abrasive surface. Based on reports, Gentlefile instruments exhibit higher cyclic fatigue resistance and smaller vertical forces compared to NiTi rotary instruments. However, Gentlefile removes debris with excellent efficiency and preserves the natural anatomy of the canal (3,8).

Therefore, this study aimed to compare the effectiveness of the two rotary systems of Gentlefile and Pro Taper Universal, in removing *E. faecalis* from the infected root canals.

## Material and Methods

-Teeth collection and preparation

Forty mandibular premolar teeth with complete apex extracted for orthodontic reasons were obtained. After removing the periodontal tissue and bone on the teeth with curettes, periapical radiographs were taken and only single roots with non-calcified canals were included. The tooth crowns were cut using a diamond disk to obtain a root canal with a 15 mm length. Then, the root canals were enlarged by using a hand K-type file size 15 to 25 until reached to apical foramen and washed with distilled water. All the canals were irrigated using the solution of 17% Ethylenediaminetetraacetic acid (EDTA, Merck), 1% sodium hypochlorite solution (Sigma-Aldrich) for one minute, respectively, and distilled water for smear layer removal. Next, the apical foramen of root canals were sealed using composite resin and each tooth was placed in the Eppendorf tube containing 1.5 ml of sterile brain-heart infusion (BHI, Merck, Germany). All teeth were sterilized through autoclaving (15 min, 121°C, 15 psi) and kept in an incubator (37°C, 48 h) in order to determine any possible bacterial contamination of the teeth. After teeth sterilization, samples were randomly divided into two groups: Gentlefile (MBI Tornado, France; n=18) and Pro Taper Universal (Dentsply Maillefer, Baillagues, Switzerland; n=18). Besides, four teeth were used as a negative control.

-Root canal contamination

In this study, a pure culture suspension of *E. faecalis* (ATCC 29212, Iranian Biological Resource Center) was used for infecting the root canals. Each sterilized tooth was removed from the BHI and after a few minutes, the root canal was filled with 2 µl of *E. faecalis* suspensions (1.5 × 108 CFU/mL) using a manual single-channel pipette (0.5-10 μL, BOECO, Germany). After 5 min, the teeth was placed into the Eppendorf tube again, and then was incubated at 37°C for 4 weeks. Every week, BHI was substituted with fresh medium in the Eppendorf tube under sterile conditions.

-Root canal instrumentation

Before and after the instrumentation of the root canals, a piece of #20 sterile paper point (Diadent, Korea) was placed and retained for the 30s in the canal. The collected samples were transferred to the Eppendorf tubes containing 1mL BHI, and then vortexed for the 30s. A 10× serial dilution was performed and 100 µl of each sample was cultivated on BHI agar using the spread plate method. After incubation at 37°C for 24h, the number of colonies were counted as CFUs/ml.

The root canals were cleaned and shaped with Pro Taper files, following the manufacturer’s instructions and using the Crown-Down method, with SX, S1, S2, F1, and F2 files, until reaching the working length. For instrumentation of the root canals with Gentlefile system, based on the manufacturer’s instructions, grey (22#), yellow (21#), red (23#), and blue (26#) files, were used to proceed towards the working length. During the instrumentation, the canals were irrigated extensively with normal saline (0.9% NaCl, Merck, Germany) after the use of each file.

-Statistical Analysis

The data were analyzed using the statistical SPSS software (SPSS for Windows 16, SPSS Inc., Chicago, IL.). The Shapiro-Wilk test was used for the normality of the variables’ distribution. The paired t-test was used to investigate the differences in the CFU of *E. faecalis* obtained before and after the instrumentation. The Student’s t-test was performed for means comparison.

The level of significance for all analysis was *p*<0.05.

## Results

According to the results, all sterilized teeth and negative group were shown to be negative for any contamination. Although two rotary systems did not completely eliminate *E. faecalis* from the root canals, the bacterial decrease percentage value of Gentlefile and Pro Taper Universal were 96.1% and 90%, respectively. As the findings of this study illustrated in Table 1 show, Gentlefile group was significantly (*p*<0.001) more effective in decreasing bacterial populations compared to Pro Taper Universal group.

**Table 1:**
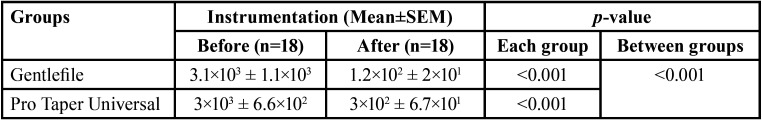
Effectiveness of Gentlefile and Pro Taper Universal systems for the removal of cultivable *E. faecalis* (CFU/ml) from infected root canals.

## Discussion

Our results clearly demonstrated that the number of bacteria in infected root canals were reduced in two rotary systems; yet, they did not completely eradicate the microbial load during root canal treatment. This result agrees with other studies (9,10) which seems to be caused by untouched apical and middle areas after rotary system instrumentation (10). A study reported that 35% or more of the canals’ surface area stayed still intact after NiTi rotary techniques (11). Htun *et al*. (2020) estimated the percentages of the untouched apical and middle areas to be roughly around 11% after instrumentation. On the other hand, their study indicated that the Gentlefile and NiTi rotary systems do not differ in terms of creating unprepared surfaces in canals (9). Also, the studies based on micro-computed tomography (micro-CT) and scanning electron microscopy (SEM) reported that instruments have been shown to not reach some parts of the canal areas; so, these regions remained untouched (12,13).

To our knowledge, there are no comparative information available on the cleaning effectiveness of the two Gentlefile and Pro Taper Universal systems. In the present study, the Gentlefile system was demonstrated significantly more effective in reducing bacterial populations than Pro Taper Universal system. In the present study, ProTaper Universal system showed the lowest bacterial reduction, which is in accordance with previous studies (14,15), possibly because it has a symmetrical design and constant pitch. A study by Capar *et al*. (2014) showed that cross-sectional design and apical taper of rotary instruments could have led to a larger quantity of debris being extruded apically (16). Tewari *et al*. (2016) stated that the ultra-flexibility of the instruments could influence the bacterial reduction because of their cutting power which deforms easily in the root canal walls against slight pressure (17). Gentlefile system, as an ultra-flexible instrument, exhibited higher smear layer and less remaining debris that helps with reaching better cleaning efficacy and decreased reinfection risk (18). Also, these instruments cannot cut into the dentin but rather abrade the root canal dentin walls compared to Pro Taper Universal system (9). Moreover, it has been reported that NiTi instruments cannot achieve complete canal cleanliness, especially in the apical area (11,19). Even so, our results contradict the report obtained by Neelakantan who found no significant difference in root canal debridement between Gentlefile and NiTi rotary instruments (20). This disagreement could be resulted from the different methodology they have applied including histologic examination of the remaining pulp tissues.

## Conclusions

The two rotary systems of Gentlefile and Pro Taper Universal are highly effective in bacterial reduction from root canals while Gentlefile system showing greater cleanliness than those instrumented with Pro Taper Universal system.
